# hPMSCs-Derived Exosomal miRNA-21 Protects Against Aging-Related Oxidative Damage of CD4^+^ T Cells by Targeting the PTEN/PI3K-Nrf2 Axis

**DOI:** 10.3389/fimmu.2021.780897

**Published:** 2021-11-23

**Authors:** Yanlian Xiong, Yanlei Xiong, Hengchao Zhang, Yaxuan Zhao, Kaiyue Han, Jiashen Zhang, Dongmei Zhao, Zhenhai Yu, Ziran Geng, Longfei Wang, Yueming Wang, Xiying Luan

**Affiliations:** ^1^ Department of Anatomy, School of Basic Medicine, Binzhou Medical University, Yantai, China; ^2^ Department of Pathology, Xuanwu Hospital, Capital Medical University, Beijing, China; ^3^ Department of Immunology, School of Basic Medicine, Binzhou Medical University, Yantai, China

**Keywords:** aging, miR-21, Nrf2, CD4 + T cells, hPMSC, exosomes

## Abstract

Mesenchymal stem cells (MSCs)-derived exosomes were considered a novel therapeutic approach in many aging-related diseases. This study aimed to clarify the protective effects of human placenta MSCs-derived exosomes (hPMSC-Exo) in aging-related CD4^+^ T cell senescence and identified the underlying mechanisms using a D-gal induced mouse aging model. Senescent T cells were detected SA-β-gal stain. The degree of DNA damage was evaluated by detecting the level of 8-OH-dG. The superoxide dismutase (SOD) and total antioxidant capacity (T-AOC) activities were measured. The expression of aging-related proteins and senescence-associated secretory phenotype (SASP) were detected by Western blot and RT-PCR. We found that hPMSC-Exo treatment markedly decreased oxidative stress damage (ROS and 8-OH-dG), SA-β-gal positive cell number, aging-related protein expression (p53 and γ-H2AX), and SASP expression (IL-6 and OPN) in senescent CD4^+^ T cells. Additionally, hPMSC-Exo containing miR-21 effectively downregulated the expression of PTEN, increased p-PI3K and p-AKT expression, and Nrf2 nuclear translocation and the expression of downstream target genes (NQO1 and HO-1) in senescent CD4^+^ T cells. Furthermore, *in vitro* studies uncovered that hPMSC-Exo attenuated CD4^+^ T cell senescence by improving the PTEN/PI3K-Nrf2 axis by using the PTEN inhibitor bpV (HOpic). We also validated that PTEN was a target of miR-21 by using a luciferase reporter assay.** **Collectively, the obtained results suggested that hPMSC-Exo attenuates CD4^+^ T cells senescence *via* carrying miRNA-21 and activating PTEN/PI3K-Nrf2 axis mediated exogenous antioxidant defenses.

## Introduction

Immunosenescence is accompanied by the dysfunction of T cells, leading to age-related immune dysfunction and increases the susceptibility to infectious and cancer diseases (1, 2). Recent evidence suggests that CD4^+^ T cell senescence is characterized by reactive oxygen species (ROS) activation, leading to irreversible proliferation arrest (3, 4). Recently, the role of mesenchymal stem cells (MSCs) in aging progression has aroused widespread attention. Currently, our laboratory have found that placenta-derived mesenchymal stromal cells (hPMSCs) attenuate D-gal induced CD4^+^ T cell senescence by targeting nuclear factor erythroid-2-related factor 2 (Nrf2)-mediated antioxidant defenses (5). However, the direct link between hPMSCs intervention and activation of the Nrf2 pathway in senescent CD4^+^ T cells remains to be elucidated.

Exosomes, a subset of extracellular vesicles (EVs), function as a mode of molecular transfer and intercellular communication, which facilitate the direct extracellular transfer of cytokines and growth factors, lipids, and RNA/DNAs between cells (6, 7). Recently, MSC exosomes are gaining attention in the treatment of various aging-related diseases (8). Han et al. have demonstrated that MSC derived exosomes prevent aging-related vascular dysfunction in mouse hindlimb (9). Zhu et al. have reported that UMSC derived exosomes prevent aging-induced cardiac dysfunction (10). However, whether MSC-derived exosomes also contribute to the protection of the CD4^+^ T cell senescence needs to be further explored.

miR-21 has been suggested to play a vital role in various pathological and biological processes, including cell survival, inflammation, and apoptosis (11). Recently, miR-21 is considered to be involved in the metabolic processes of CD4^+^ T cells activation, proliferation and apoptosis in different diseases (12). Dong et al. reported that decreased expression of miR-21 in CD4^+^ T cells from rheumatoid arthritis patients compared to controls, and the downregulated miR-21 showing a strong correlation with an improved ratio of Th17/Treg cells (13). A variety of target genes has been reported to be regulated by miRNA-21. Among the target genes of miR-21, the Phosphatase and tensin homolog deleted on chromosome 10/phosphoinositide 3-kinase/protein kinase B (PTEN/PI3K/Akt) signaling pathway has been implicated as an important regulator of the proliferation, differentiation, and apoptosis of a variety of cells (14, 15). Interestingly, our previous results indicated that hPMSCs weaken CD4^+^ T cell senescence by activating the Akt-mediated Nrf2 antioxidant signaling (5). However, the protection effect and mechanism of hPMSCs derived exosomes (hPMSC-Exo) on aging-related T cell dysfunction remain unknown.

Therefore, in the current study, we aim to explore the roles of exosomes with miR-21 from hPMSCs could attenuate aging-related T cell damage and dysfunction, thereby clinically alleviating immunosenescence.

## Materials And Methods

### Reagents

DAPI(#4083) and antibodies against Histon H3 (#4499), Nrf2 (#12721), PI3K (#4249), Akt (#9272), β-actin (#4970), HO-1 (#43966), p53 (#2527), p-PI3K (#17366), p-Akt (#4060) and NQO1 (#62262) were obtained from CST corporation (Massachusetts, USA). bpV(HOpic) (SML0884), anti-γ-H2AX (05-636-AF555), and PKH26 (PKH26PCL) were obtained from Sigma-Aldrich (St. Louis., MO, USA). 8-Hydroxy-2’-deoxyguanosine(8-OH-dG) kit (SKT-120-96S) was obtained from StressMarq Biosciences (Victoria, BC, Canada). SOD kit (BC0170) and Total antioxidant capacity (T-AOC) kit (BC1310) were obtained from Solarbio (Qingdao, China). RNA was extracted using a RNeasy Plus Mini kit obtained from Qiagen (#74134, Qiagen Inc, CA, USA).

### Animal Models

Male C57BL/6 mice (8 weeks) were obtained from Lvye Pharmaceutical Co., Ltd. Shandong, China (SYXK 2018–0028). Mice were free access to water and chow diet and housed in a standard environment with a regular 12/12 h light/dark cycle. This study was performed following protocols approved by the Binzhou Medical University Institutional Animal Care and Use Committee (permit number: 2020-06).

The mice were randomly divided into four groups (n = 10): (1) control group, treated with saline (20 mL kg^-1^ day^-1^) as a vehicle for 7 weeks; (2) D-gal group, treated with D-gal (200 mg kg^-1^ day^-1^) for 7 weeks; and (3) hPMSC-Exo group, D-gal modeling followed by 100 µg hPMSC-Exo week^-1^ intervention (from 5 to 7 week); (4) PBS group, D-gal modeling followed by150 μL PBS kg^-1^ week^-1^ intervention (from 5 to 7 week). The detailed experimental scheme is exhibited in **Figure 2A**. CD4^+^ T cells were isolated from single-cell suspensions of splenocytes using a magnetic cell separator as described previously (16).

### Isolation of hPMSCs

As described previously (17), hPMSCs were isolated from human term placentas of donors. Isolated hPMSCs were identified by the immunophenotype for human leukocyte antigens HLA-DR, CD90, CD14, CD105, CD19, CD34, and CD73 using flow cytometry (FCM) and observed the cells morphology using microscopy. The hPMSCs were cultured in osteogenic and adipogenic induced medium to differentiate into osteoblasts and adipocytes and followed Alizarin Red staining to verify osteogenesis differentiation and Oil Red O staining to verify adipogenic differentiation.

### Isolation and Purification of hPMSC-Exo

hPMSC-derived exosomes were harvested from passage 3 MSCs grown to 80% confluency in 10-cm plates (Corning) using the VEX Exosome Isolation Reagent (R601, Vazyme Biotech) as described previously (18). In short, the collected hPMSCs culture supernatant was centrifuged at 200 ×g for 30 min at 4°C. After being mixed with exosome isolation reagent overnight at 4°C, the collected supernatant was centrifuged at 10,000 ×g for 1 h and collect the pellet to resuspended in 200 μl of PBS and stored at -80°C or used immediately.

Isolated exosomes were identified by transmission electron microscopy (TEM) (JEOL JEM-1230), western blotting of exosomal surface marker proteins (CD63, CD9, and HSP70, and use Cytochrome-C (Cyt-c) as a negative control), and NanoSight analysis (Malvern Instruments, Malvern, UK).

### Naive CD4^+^ T Cell Isolation and Co-Culture With hPMSC-Exo

Human naive CD4^+^ T (CD4CD45RA) cells were isolated by CD4^+^ T Cell Isolation Kit II (Miltenyi, Bergisch Gladbach, Germany). Cells were pretreated with the PTEN inhibitor bpV (HOpic)(100 μM) for 1 h. To over-express or inhibit miR-21 in hPMSCs, miR-21 inhibitor, inhibitor NC, mimics NC, and miR-21 mimics (GenePharma Co. Ltd., Shanghai, China) were used to treat the hPMSCs. By using the IL-2 (2.5 ng/ml) and anti-CD3/CD28 Dynabeads (1 μg/ml) as mitogenic stimulus, 2 µg/ml hPMSC-Exo were added to CD4^+^ T cells (4×10^6^) and cultured at 37°C in 5% CO_2_ for 72 h.

Purified hPMSC-Exo were incubated with PKH26 (Sigma-Aldrich) for 5 min at room temperature. The PKH26 labeled hPMSC-Exo were resuspended in PBS and incubated with CD4^+^ T cells at 37°C for 6 h. After counter-stained nuclei with DAPI, the fluorescence image was captured using a laser scanning confocal microscope (FV3000, Olympus Corporation, Japan).

### Antioxidant Biomarkers Detection

The CD4^+^ T cells of each group were harvested and lysed by ultrasonication in the presence of a protease inhibitor. The supernatant was collected for analysis after centrifugation at 3,000 × g for 10 min. The levels of T-AOC and SOD in the supernatant were measured using appropriate kits following the manufacturer’s instructions (Solarbio, Qingdao, China).

### Oxidative Stress Biomarkers Detection

The intracellular production of ROS was assessed by 2’,7’-dichlorofluorescein diacetate (H2DCF-DA) (Sigma, St Louis, MO, USA) (19). The detection procedure was carried out following the instructions provided by the manufacturer.

The CD4^+^ T cells were re-suspended and the DNA was isolated using the method recommended by ESCODD. The 8-OH-dG in the DNA was detected using an ESA Coulochem II electrochemical detector in line with a UV detector as previously described (20).

### SA-β-Gal Staining

The percentage of SA-β-Gal positive CD4^+^ T cells was measured as described previously (21). In short, CD4^+^ T cells were incubated with SA-β-Gal staining solution overnight at 37°C after being fixed with 3% formaldehyde. After washing with PBS, SA-β-Gal positive CD4^+^ T cells were determined under microscopy (Leica, Germany).

### Western Blot Analysis

Western blot analyses were performed as described previously (22). Briefly, after SDS-PAGE, proteins were electro-transferred to PVDF membranes and blocked in 5% BSA dissolved in TBST for 2h at room temperature. After incubated with primary antibody overnight at 4°C, PVDF membranes were incubated with secondary antibody 2 h at room temperature. The protein bands were visualized using developer (NCI4106, Pierce, Rockford, IL, USA) and performed densitometric analysis by the software.

### Dual-Luciferase Reporter Gene Assay

The potential binding site between PTEN and miR-21 was identified using TargetScan (http://www.targetscan.org). The miR-21 mimics/inhibitor and corresponding negative control (NC) were synthesized by Guangzhou RiboBio Co., Ltd. Amplicons were inserted between SacI and XbaI cleavage sites of pmirGLO vector (Promega, U.S.A.). The human embryonic kidney cell line (HEK) 293T cells (purchased from Procell Life Science & Technology Co., Ltd., Wuhan, China) were selected based on the low endogenous miRNA expression. 800 ng wild-type or mutant reporter and 20 μM miR-21 mimic, inhibitor (GenePharma Co., Ltd, China) were co-transfected into HEK293T cells using Lipofectamine 2000 (Invitrogen, U.S.A.). 24 h after transfection, Renilla and Firefly luciferase activity of lysed HEK293T cells was determined by Dual-Luciferase Reporter Assay System (Promega).

### RNA Extraction and Quantitative Real-Time PCR

miRNA and mRNA was measured using a quantitative real-time PCR assay. The relative abundance of genes was calculated using the 2^-ΔΔCT^ formula. U6 served as the internal reference for miR-21, and β-actin was the internal reference of mRNA. Primers are attached in **Additional File 1**.

### Statistical Analysis

Data were expressed as mean ± SEM. Statistical significance is determined by unpaired two-tailed Student’s t-test (or nonparametric test), one-way ANOVA followed by the Tukey’s test for multiple comparisons (23). A value of *P*< 0.05 was statistically significant.

## Results

### Characterizations of hPMSCs and hPMSCs-Secreted Exosomes

**Figure 1:**
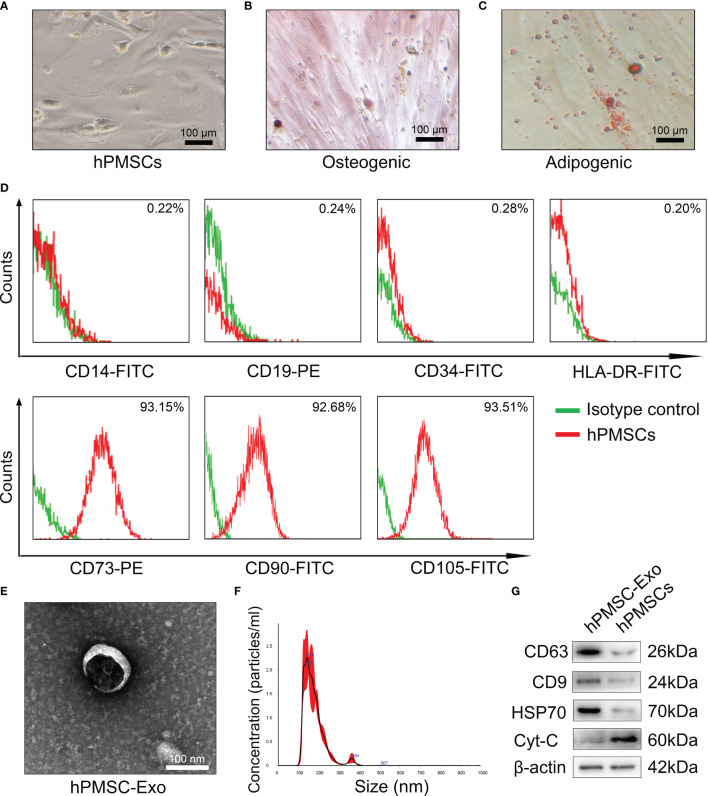
Characterizations of hPMSCs and hPMSCs-secreted exosomes. **(A)** The typical fibroblastic morphology of hPMSCs was verified by microscope. **(B)** Alizarin Red staining to verify osteogenesis differentiation of hPMSCs. **(C)** Oil Red O staining to verify adipogenic differentiation of hPMSCs (Bar =100 µm). **(D)** The surface markers of hPMSCs were tested by FCM. **(E)** The morphology of hPMSC-Exo under an electron microscope. (Bar =100 nm). **(F)** NTA to determine the diameter of hPMSC-Exo. **(G)** Positive exosome markers CD63, CD9, and HSP70 and negative exosome markers Cyt-C were evaluated by western blotting.

As shown in **Figure 1A**, the hPMSCs showed typical fibroblastic morphology. The hPMSCs were cultured in adipogenic and osteogenic induction medium to differentiate into osteoblasts(**Figure 1B**) and adipocytes (**Figure 1C**), respectively. In addition, the FCM results suggested that more than 95% of isolated hPMSCs expressed CD105, CD90, CD73 and but not HLA-DR, CD19, CD14, or CD34 (**Figure 1D**).

Electron microscopy exhibited revealed typical circular particles ranging from 30 to 200 nm in diameter, and NTA revealed a similar size distribution of hPMSC-Exo (**Figures 1E, F**). Western blot analysis showed that hPMSC-Exo were positive for CD63, CD9, and HSP70 (positive markers for exosomes), but negative for Cyt-C (negative markers for exosomes) (**Figure 1G**).

### hPMSC-Exo Attenuates D-Gal-Induced CD4^+^ T Cells Senescence in Mice

**Figure 2:**
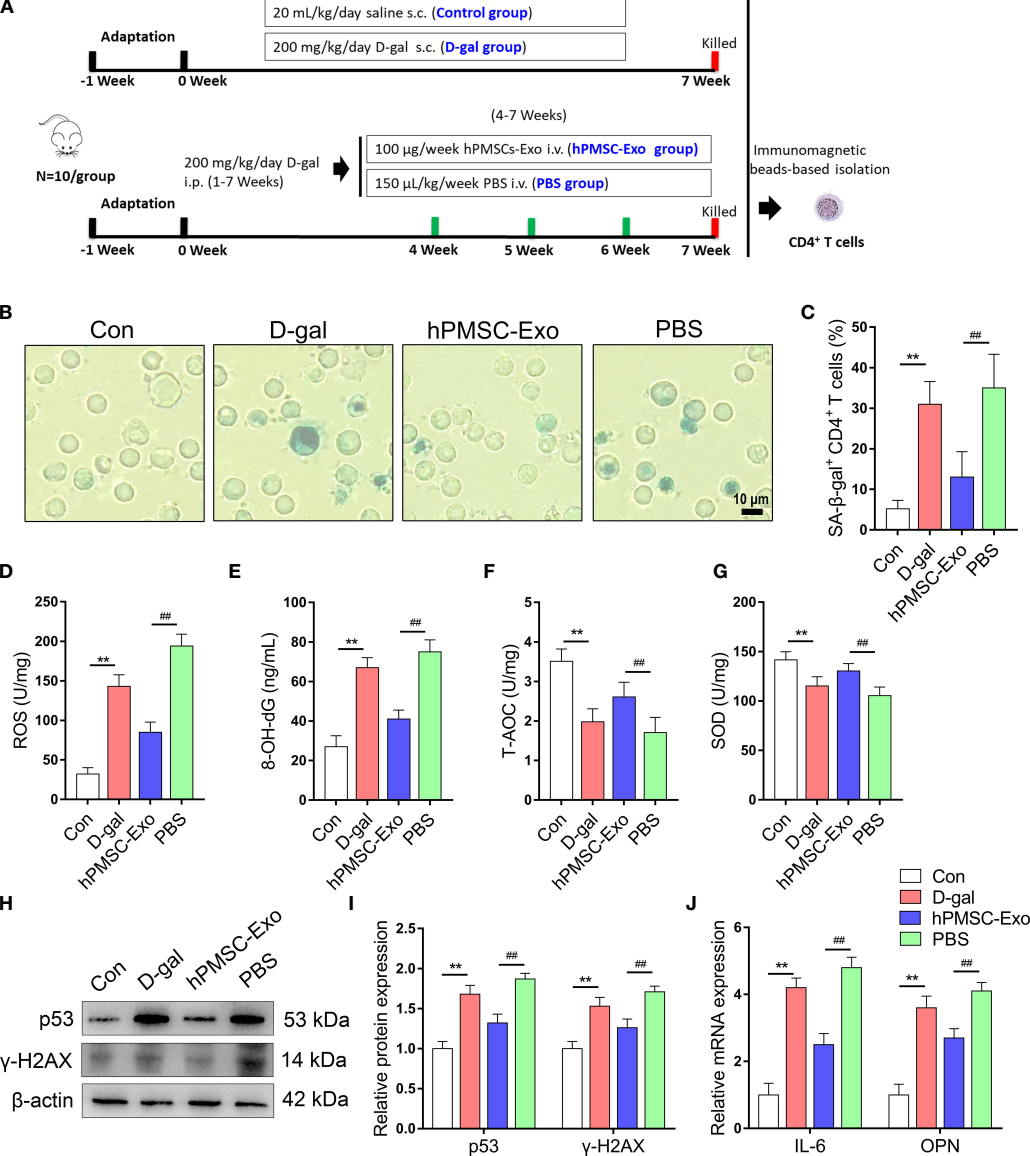
hPMSC-Exo attenuates D-gal-induced CD4^+^ T cells senescence in mice. **(A*)*** The scheme of the experimental design. **(B, C)** The rate of SA-β-gal-positive CD4^+^ T cells (Bar =10 µm). **(D, E)** The levels of ROS and 8-OH-dG. **(F, G)** The levels of T-AOC and SOD activity. **(H, I)** The protein expressions of senescent markers p53 and γ-H2AX. **(J)** The levels of OPN and IL-6 mRNA. Data represent the mean scores ± SEM of at least three independent experiments. **p < 0.01 vs. control group; ^##^ *p* < 0.01 *vs*. PBS group.

The animal experiment schematic of hPMSC-Exo intervention is shown in **Figure 2A**. We purified CD4^+^ T cells using immunomagnetic beads and tested the SA-β-gal positive rate of CD4^+^ T cells. As shown in **Figures 2B, C**, the rate of SA-β-gal-positive CD4^+^ T cells significantly improved in the D-gal group compared with the control group. Conversely, the rate of SA-β-gal-positive CD4^+^ T cells was significantly reduced in hPMSC-Exo group compared with the PBS group (**Figures 2B, C**).

To evaluate the effects of hPMSC-Exo treatment on the redox metabolism of CD4^+^ T cells during aging, we detected the change of oxidative stress biomarkers and antioxidant capacity in CD4^+^ T cells. The ROS and 8-OH-dG levels of CD4^+^ T cells markedly improved in the D-gal group compared with the control group, while hPMSC-Exo intervention markedly reduced the levels of ROS and 8-OH-dG compared with the PBS group (**Figures 2D, E**). In contrast, the activities of T-AOC and SOD declined markedly in the D-gal group compared with the control group, while hPMSC-Exo intervention significantly increased the activities of T-AOC and SOD compared with the PBS group (**Figures 2F, G**).

In addition, the aging-related protein expression of p53 and γ-H2AX markedly improved in the D-gal group compared with the control group, while hPMSC-Exo intervention significantly reduced the levels of p53 and γ-H2AX compared with the PBS group (**Figures 2H, I**).

Secretion of pro-inflammatory factors is considered to be a key feature of the SASP phenotype, including OPN, IL-6, and proinflammatory chemokines (24, 25). As shown in **Figure 2J**, D-gal treatment markedly increased the mRNA levels of IL-6 and OPN compared with the control group, while hPMSC-Exo intervention significantly reduced the levels of IL-6 and OPN compared with the PBS group. These results suggested that hPMSC-Exo treatment weakens CD4^+^ T cell senescence in aging mice.

### hPMSC-Exo Promotes the Expression of PTEN/PI3K-Nrf2 Signaling Pathway

**Figure 3:**
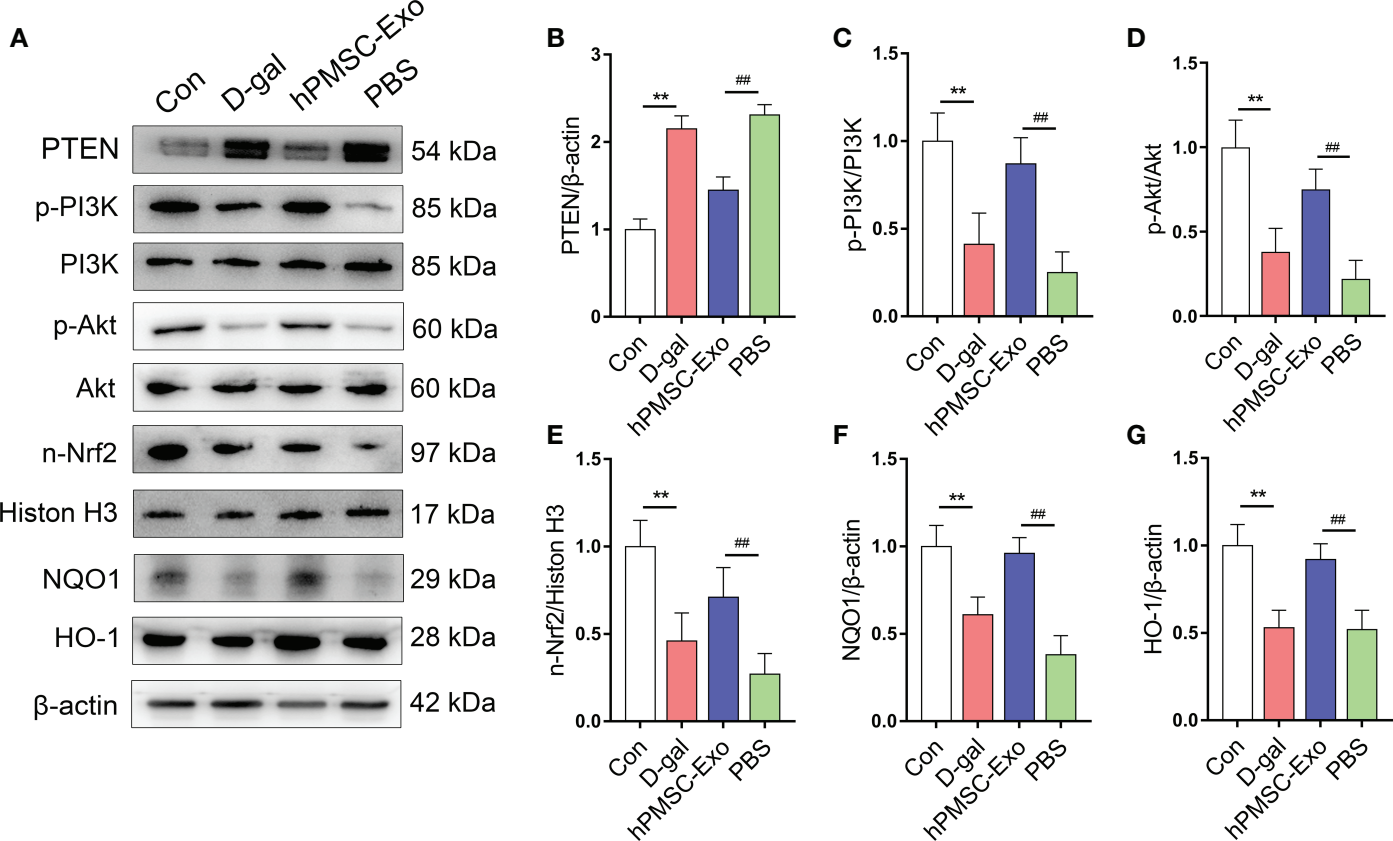
hPMSC-Exo upregulates the expression of Nrf2 *via* PTEN/PI3K axis in CD4^+^ T cells from D-gal-treated mice. **(A)** The protein expressions of PTEN/PI3K-Nrf2 axis and its downstream target genes were evaluated by Western blot. **(B)** The protein level of PTEN. **(C)** The ratio of p-PI3K/PI3K. **(D)** The ratio of p-Akt/Akt. **(E)** The protein level of nuclear Nrf2. **(F)** The protein level of NQO1. **(G)** The protein level of HO-1. Data represent the mean scores ± SEM of at least three independent experiments. **p < 0.01 *vs*. control group; ^##^ *p* < 0.01 *vs*. PBS group.

Nrf2 plays a crucial role in regulating intracellular redox balance, senescence, and inflammation (26, 27). Previous research suggested that the activation of Nrf2 is regulated by triggering PTEN/PI3K-mediated degradation and nuclear export of Nrf2 (27). Here, we found markedly increased expression of PTEN and declined phosphorylation of PI3K and Akt in CD4^+^ T cells from the D-gal group compared with the control group (**Figures 3A–D**). While hPMSC-Exo intervention markedly decreased the expression of PTEN and markedly raised the phosphorylation of PI3K and Akt in CD4^+^ T cells compared with the PBS intervention group (**Figures 3A–D**).

Furthermore, we found that the expression of nuclear Nrf2 and downstream target genes HO-1 and NQO1 were significantly declining in CD4^+^ T cells from the D-gal group compared with the control group (**Figures 3E–G**). Conversely, hPMSC-Exo intervention markedly improved the expression of these antioxidant proteins in comparison with that of the PBS intervention group (**Figures 3E–G**). These data indicated that hPMSCs treatment promoted the nuclear transfer of Nrf2 and the expression of Nrf2 target antioxidant genes. Therefore, the obtained data indicated that hPMSC-Exo attenuates CD4^+^ T cell senescence by targeting Nrf2 functions *via* PTEN/PI3K pathway.

### miR-21 Targets and Negatively Regulates PTEN

**Figure 4:**
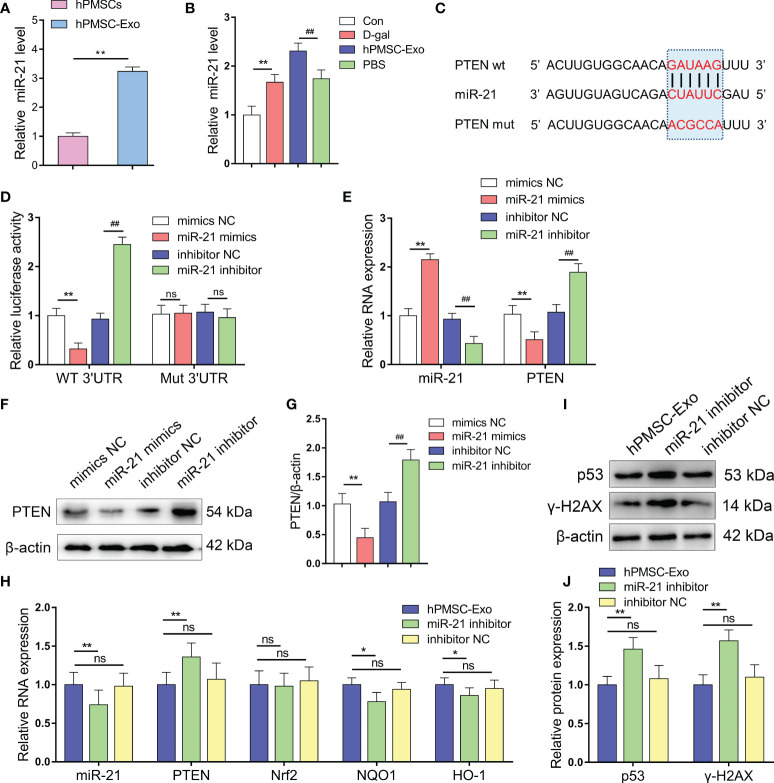
miR-21 targets and negatively regulates PTEN. **(A)** The expression of miR-21 in hPMSCs and hPMSC-Exo. **(B)** The expression of miR-21 in CD4+ T cells. **(C)** Binding site prediction of miR-21 in PTEN 3'-UTR using TargetScan. **(D)** The luciferase activity was measured by a dual-luciferase reporter gene assay. **(E)** The levels of miR-21 and PTEN mRNA in 293T cells after miR-21 overexpression and inhibition. **(F–G)** PTEN protein level after miR-21 overexpression and inhibition. **(H)** The RNA levels of miR-21, PTEN, Nrf2, NQO1, and HO-1 in the CD4+ T cells of D-gal treated mice after hPMSC-Exo, miR-21 inhibitor treated hPMSC-Exo, and inhibitor NC treated hPMSC-Exo treatment. **(I–J)** The protein expressions of senescent markers p53 and γ-H2AX in the CD4+ T cells of D-gal treated mice after hPMSC-Exo, miR-21 inhibitor treated hPMSC-Exo, and inhibitor NC treated hPMSC-Exo treatment. Data represent the mean scores ± SEM of at least three independent experiments. *p < 0.05; **p < 0.01; ^##^p < 0.01; ns means p > 0.05.

We found that miR-21 is expressed at a significantly higher level in hPMSC-Exo than in hPMSCs themselves, suggesting that MSCs package the majority of the miR-21 produced in exosomes (**Figure 4A**). In addition, we found markedly increased expression of miR-21 in CD4^+^ T cells from the D-gal group compared with the control group. Conversely, hPMSC-Exo intervention further increased the expression of miR-21 in CD4^+^ T cells compared with the PBS intervention group (**Figure 4B**).

To validate whether PTEN is a target gene of miR-21, we performed a dual-luciferase reporter assay and identified the miR-21 binding site in PTEN, which was predicted by TargetScan to be in the 3’-UTR (**Figure 4C**). miR-21 mimics treatment downregulated the luciferase activity compared to the control group, whereas miR-21 inhibitor treatment markedly improved the relative luciferase activity. In addition, the effect of miR-21 on luciferase activity was not detected when the seed sequence of the miR-21 binding sites was mutated (**Figure 4D**). In addition, we modified the expression of miR-21 in 293T cells and using qRT-PCR and Western blot analysis demonstrated decreased PTEN mRNA and protein levels in cells treated with miR-21 mimics, while opposite trends were observed in cells treated with miR-21 inhibitor (**Figures 4E–G**). These data indicated that PTEN was targeted and negatively regulated by miR-21.

In the CD4^+^ T cells of D-gal treated mice, the level of miR-21was significantly decreased in the hPMSC-Exo miR-21 inhibitor treatment group compared with hPMSC-Exo group (**Figure 4H**). Furthermore, the miRNA level of PTEN was markedly increased, while the mRNA levels of HO-1 and NQO1 were significantly decreased after hPMSC-Exo miR-21 inhibitor treatment (**Figure 4H**). In addition, the aging-related protein expression of p53 and γ-H2AX were also improved significantly in the hPMSC-Exo miR-21 inhibitor treatment group compared with hPMSC-Exo group (**Figures 4I, J**). These results suggested hPMSCs ameliorate CD4^+^ T cells senescence by exosomes transmitted miR-21.

### hPMSC-Exo miR-21 Inhibits PTEN Expression in CD4^+^ T Cells

**Figure 5:**
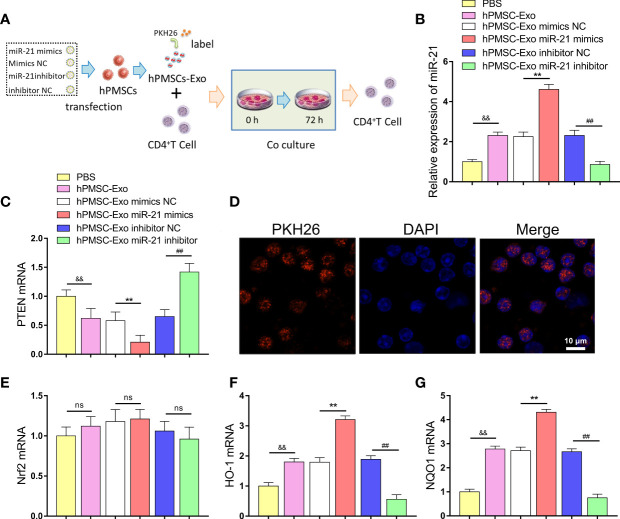
hPMSCs transfer miR-21 to CD4^+^T cells through exosomes to repress the expression of PTEN. **(A)** The scheme showing the experimental design. **(B)** The expression of miR-21 in CD4^+^T cells after co-cultured with hPMSC-Exo. **(C)** Expression of PTEN in CD4^+^T cells co-cultured with hPMSC-Exo. **(D)** The PKH26-labeled hPMSC-Exo was internalized by CD4^+^T cells (Bar =10 µm). **(E–G)** Expression of Nrf2, NQO1, and HO-1 in CD4^+^T cells co-cultured with hPMSC-Exo. Data represent the mean scores ± SEM of at least three independent experiments. **p < 0.01 vs. mimics NC; ^##^ *p* < 0.01 vs. inhibitor NC; ^&&^ *p* < 0.01 vs. PBS group. ns means p > 0.05.

We further investigated the regulatory effect of hPMSC-Exo miR-21 on PTEN expression in CD4^+^ T cells (**Figure 5A**). The exosomes treated with different plasmids were co-cultured with CD4^+^ T cells, and the expression of miR-21 and PTEN was detected. The expression of miR-21 was markedly improved in CD4^+^ T cells after co-cultured with hPMSC-Exo compared PBS treatment group (**Figure 5B**). Conversely, hPMSC-Exo treatment greatly decreased the expression of PTEN in CD4^+^ T cells compared with the PBS group (**Figure 5C**). Moreover, hPMSC-Exo treated with miR-21 mimics further increasing the expression of miR-21 and decreasing the expression of PTEN in CD4^+^ T cells compared hPMSC-Exo treatment group, while the opposite trend appeared in the hPMSC-Exo treatment miR-21inhibitor group (**Figures 5B, C**). After incubation with CD4^+^ T cells, PKH26-labeled hPMSC-Exo exhibited red fluorescence in the cytoplasm of CD4^+^ T cells, implying the hPMSC-Exo uptake by CD4^+^ T cells (**Figure 5D**).

In addition, we further explored the effect of hPMSC-Exo on Nrf2 and downstream target antioxidant genes NQO1 and HO-1 expression in CD4^+^ T cells. Although hPMSC-Exo treatment did not cause significant changes in Nrf2 expression, the expression of NQO1 and HO-1 was significantly increased in CD4^+^ T cells compared PBS treatment group (**Figures 5E–G**). Moreover, hPMSC-Exo treated with miR-21 mimics further upregulating the expression of NQO1 and HO-1 in CD4^+^ T cells compared hPMSC-Exo treatment group, while the opposite trend appeared in the hPMSC-Exo treatment miR-21 inhibitor group (**Figures 5F, G**). The aforementioned results suggested that hPMSC-Exo was critical in transferring exogenous miR-21 from hPMSCs to CD4^+^ T cells and regulate the expression of PTEN/PI3K-Nrf2 signaling pathway.

### hPMSC-Exo Upregulates the Expression of PTEN/PI3K-Nrf2 Signaling Pathway in Senescent CD4^+^ T Cells

**Figure 6:**
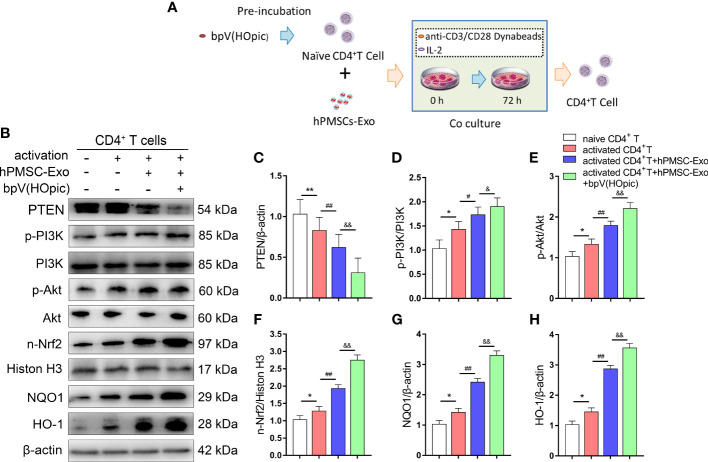
hPMSC-Exo upregulates the expression of Nrf2-regulated antioxidant genes in senescent CD4^+^ T cells. 4×10^6^ human naive CD4^+^ T cells (CD4CD45RA cells) were pretreated for 1 h with the PTEN inhibitor bpV(HOpic)(100 μM). Then, 2 µg/ml hPMSC-Exo were added to CD4^+^ T cells and co-cultured for 72 h. **(A)** The scheme showing the experimental design. **(B)** The protein expressions of PTEN/PI3K-Nrf2 axis and its downstream target genes in CD4^+^ T cells. **(C)** The protein level of PTEN. **(D)** The ratio of p-PI3K/PI3K. **(E)** The ratio of p-Akt/Akt. **(F)** The protein level of nuclear Nrf2. **(G)** The protein level of NQO1. **(H)** The protein level of HO-1. Data represent the mean scores ± SEM of at least three independent experiments. *p < 0.05, **p < 0.01 *vs*. naive CD4^+^ T cells group; ^#^ *p* < 0.05, ^##^ *p* < 0.01 *vs*. activated CD4^+^ T cells group; ^&^ *p* < 0.05, ^&&^ *p* < 0.01 *vs*. activated CD4^+^ T cells group treated with hPMSC-Exo group.

We further confirmed whether the PTEN/PI3K-Nrf2 axis plays a vital role in the protective effects observed in senescent CD4^+^ T cells treated with hPMSC-Exo. After immuno-magnetic separation, a total of 4×10^6^ human naive CD4^+^ T cells (CD4CD45RA cells) were pretreated for 1 h with the PTEN inhibitor bpV(HOpic)(100 μM). Then, 2 µg/ml hPMSC-Exo were added to CD4^+^ T cells and co-cultured for 72 h. The schematic of experimental design is shown in **Figure 6A**. The expression of PTEN significantly decreased in activated CD4^+^ T cells after cultured for 72 h, while hPMSC-Exo intervention further decreased the expression of PTEN in activated CD4^+^ T cells (**Figures 6B, C**). Furthermore, the phosphorylation of PI3K and Akt were significantly increased in activated CD4^+^ T cells, and the hPMSC-Exo intervention further improved the phosphorylation of PI3K and Akt (**Figures 6D, E**). In addition, we found that the expression of nuclear Nrf2 and downstream target antioxidant genes NQO1 and HO-1 was markedly improved in activated CD4^+^ T cells, and the hPMSC-Exo treatment further improved the expression of these proteins (**Figures 6F–H**). Moreover, significantly improve nuclear Nrf2, NQO1, and HO-1 expression were also found in hPMSC-Exo treated CD4^+^ T cells after bpV(HOpic) supplementation (**Figures 6F–H**). These results suggested that hPMSC-Exo promotes the PTEN/PI3K-Nrf2 pathway activation by inhibiting PTEN in senescent CD4^+^ T cells.

### Inhibition of PTEN/PI3K Pathway Improves the Protective Effects of hPMSC-Exo in Senescent CD4^+^ T Cells

**Figure 7:**
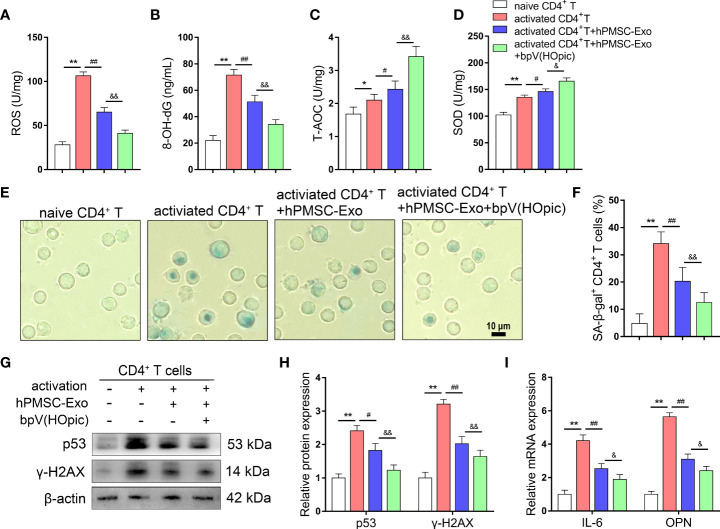
Inhibition of PTEN/PI3K pathway improves the protective effects of hPMSC-Exo in senescent CD4+ T cells. 4×10^6^ human naive CD4+ T cells (CD4CD45RA cells) were pretreated for 1 h with the PTEN inhibitor bpV(HOpic)(100 μM). Then, 2 µg/ml hPMSC-Exo were added to CD4^+^ T cells and co-cultured for 72 h. **(A, B)** The levels of ROS and 8-OH-dG. **(C, D)** The levels of T-AOC and SOD activity. **(E, F)** The rate of SA-β-gal-positive CD4^+^ T cells (Bar =10 µm). **(G, H)** The protein expressions of senescent markers p53 and γ-H2AX. **(I)** The expressions of IL-6 and OPN mRNA. Data represent the mean scores ± SEM of at least three independent experiments. *p<0.05, **p < 0.01 *vs*. naive CD4^+^ T cells group; ^#^ *p* < 0.05, ^##^ *p* < 0.01 *vs*. activated CD4^+^ T cells group; ^&^ *p* < 0.05, ^&&^ *p* < 0.01 *vs*. activated CD4^+^ T cells group treated with hPMSC-Exo group.

We further evaluated the protective effects of hPMSC-Exo on senescent CD4^+^ T cells *via* PTEN/PI3K -Nrf2 pathway, we conducted SA-β-gal staining and detected changes in redox metabolism biomarkers in CD4^+^ T cells after coculture with hPMSC-Exo. hPMSC-Exo treatment significantly decreased the levels of ROS and 8-OH-dG and the rate of SA-β-gal-positive CD4^+^ T cells in activated CD4^+^ T cells (**Figures 7A, B, E, F**). Although the activities of T-AOC and SOD were increased greatly in CD4^+^ T cells after activation, hPMSC-Exo intervention could further increase the activities of T-AOC and SOD in activated CD4^+^ T cells (**Figures 7C, D**). Furthermore, hPMSC-Exo treatment also significantly decreased the levels of aging-related protein expression of p53 and γ-H2AX and the mRNA levels of OPN and IL-6 in activated CD4^+^ T cells. Furthermore, the hPMSC-Exo mediated downregulation in the expression of aging-related mRNA and/or protein for IL-6, OPN, p53 andγ-H2AX were also enhanced by bpV(HOpic) intervention (**Figures 7G–I**). The aforementioned results suggested that the protective effects of hPMSC-Exo on senescent CD4^+^ T cells relied on PTEN/PI3K pathway mediated Nrf2 antioxidant signaling.

## Discussion

Several studies have demonstrated that aggravated oxidative damage is related to aging-induced immune dysfunction (3, 28). MSCs have been thought to be the source of seed cells for anti-aging biological therapeutics based on the immunomodulatory and antioxidant effects (29, 30). Our previous research demonstrated that hPMSCs weaken D-gal induced CD4^+^ T cell senescence *via* targeting Nrf2-mediated antioxidant defenses (5). However, due to immune rejection and limited sources, MSCs are still limited in broad clinical application. MSC-exosomes are nano-size particles released from MSCs that have been considered key carriers of paracrine factors from MSCs. Our current study shows that hPMSC-Exo carrying miR-21 could upregulate the expression of PTEN/PI3K-Nrf2 signaling pathway in senescent CD4^+^ T cells, improve the antioxidant capacity of CD4^+^ T cells, and alleviate age-related immune dysfunction.

Aging is accompanied by the deterioration of the immune system and the increase of ROS. Aggravated ROS generation in T cells will destroy the balance between oxidation and antioxidation and leads to immune dysfunction and metabolism disorder (19, 31). Here we also showed that D-gal treatment markedly improved the level of ROS, while decreased the levels of the antioxidative index (T-AOC and SOD) in CD4^+^ T cells. During aging, T cells are constantly exposed to endogenous and exogenous free radicals, which will lead to DNA damage (32). Here we also found that the levels of DNA damage and repair related indicators, such as 8-OH-dG, γ- H2AX, and p53 were increased significantly in senescent CD4^+^ T cells. In addition, senescent cells cause serious damage to various tissue microenvironments by secreting SASP, which is characterized by a significantly elevated secretion of chemokines, proinflammatory cytokines, and proangiogenic factors (33, 34). OPN is a potent proinflammatory cytokine produced by many cell types, including macrophages, dendritic cells, and activated Th1 cells, that plays a significant role in inflammatory diseases (25). Previous studies also found that the production and release of IL-6 into the circulation increased with age (35). Here we also found that the expression of OPN and IL-6 increased significantly in senescent CD4^+^ T cells. A large number of studies have shown that MSCs can regulate T cell redox metabolism *in vitro* and *in vivo*, and participate in T cell cycle regulation and subsets differentiation (36, 37). A previous study by Yan et al. suggested that hfPMSCs alleviate H_2_O_2_-induced cell apoptosis and oxidative stress by promoting the expression of Nrf2/Keap1/ARE antioxidant signaling (38). Our previous study found that hPMSCs played a protective effect by improving the Nrf2-mediated antioxidant pathway in senescent CD4^+^ T cells (5). In this study, we found that hPMSC-Exo treatment significantly increased antioxidant capacity and decreased aging-related damage and secretion phenotype in senescent CD4^+^ T cells. These results suggested that hPMSCs protect against age-associated CD4^+^ T cells senescence by transferring exosomes.

Recently, MSC-derived exosomes have been reported to play an important antioxidation, anti-apoptotic and immune-modulating role both *in vivo* and *in vitro (* 39, 40). A growing body of evidence has demonstrated that miRNAs are important intracellular regulatory molecules that are becoming the focus of studies on various diseases, especially in the regulation of immune responses (41–44). MiRNAs are differentially expressed in the cellular senescence and play important role in regulating of genes expression involved in features of ageing (45–47). Data from several studies suggest that the expression of miR-21 in the brain and heart of aged mice was significantly increased compared with adult mice (48, 49). Similarly, we also found that the expression of miR-21 was markedly increased in CD4^+^ T cells from the D-gal group. In addition, we observed that miR-21 was highly abundant in MSC-derived exosomes, and hPMSC-Exo intervention can up-regulate the expression of miR-21 in CD4^+^ T cells. Recently, miR-21 has been confirmed to negatively modulate the expression of tumor-suppressor PTEN, which is known to be an important upstream inhibitor of the PI3K/Akt signaling pathway (50, 51). Therefore, we innovatively put forward the hypothesis that hPMSCs regulate the redox metabolism of aging CD4^+^ T cells and play an anti-aging role *via* deliver miR-21 through exosomes. Here, we found that miR-21 targeted the PTEN gene in CD4^+^ T cells. In addition, hPMSC-Exo carrying miR-21 can reduce the expression of PTEN and promote the phosphorylation PI3K/Akt signaling in senescent CD4^+^ T cells. Our recent evidence suggests that hPMSCs protect against D-gal-induced CD4^+^ T cell senescence by upregulating Akt/GSK-3β/Fyn-Nrf2 pathway (5). It has been suggested that the regulation of antioxidant capacity mediated by the Nrf2 pathway is considered to be the key to the maintenance of redox metabolism during aging (52–54). Here, we showed that the capacity of miR-21 inhibitor treated hPMSC-Exo to delay the senescence of CD4^+^ T cells by regulating the expression of PTEN pathway was weakened in D-gal treated mice. *In vitro*, we found that hPMSC-Exo treated with miR-21 mimics further improving the expression of miR-21 and decreasing the expression of PTEN in CD4^+^ T cells. In addition, we observed that PTEN inhibitor treatment significantly improved the expression of nuclear Nrf2, NQO1, and HO-1 and reduced the expression of aging-related mRNA and/or protein in hPMSC-Exo treated CD4^+^ T cells. On this basis, the results of this study suggest that the trigger of the Akt/GSK-3β/Fyn-Nrf2 axis in senescent CD4^+^ T cells by hPMSCs may be achieved through the transfer of miR-21 *via* exosomes.

## Conclusions

**Figure 8:**
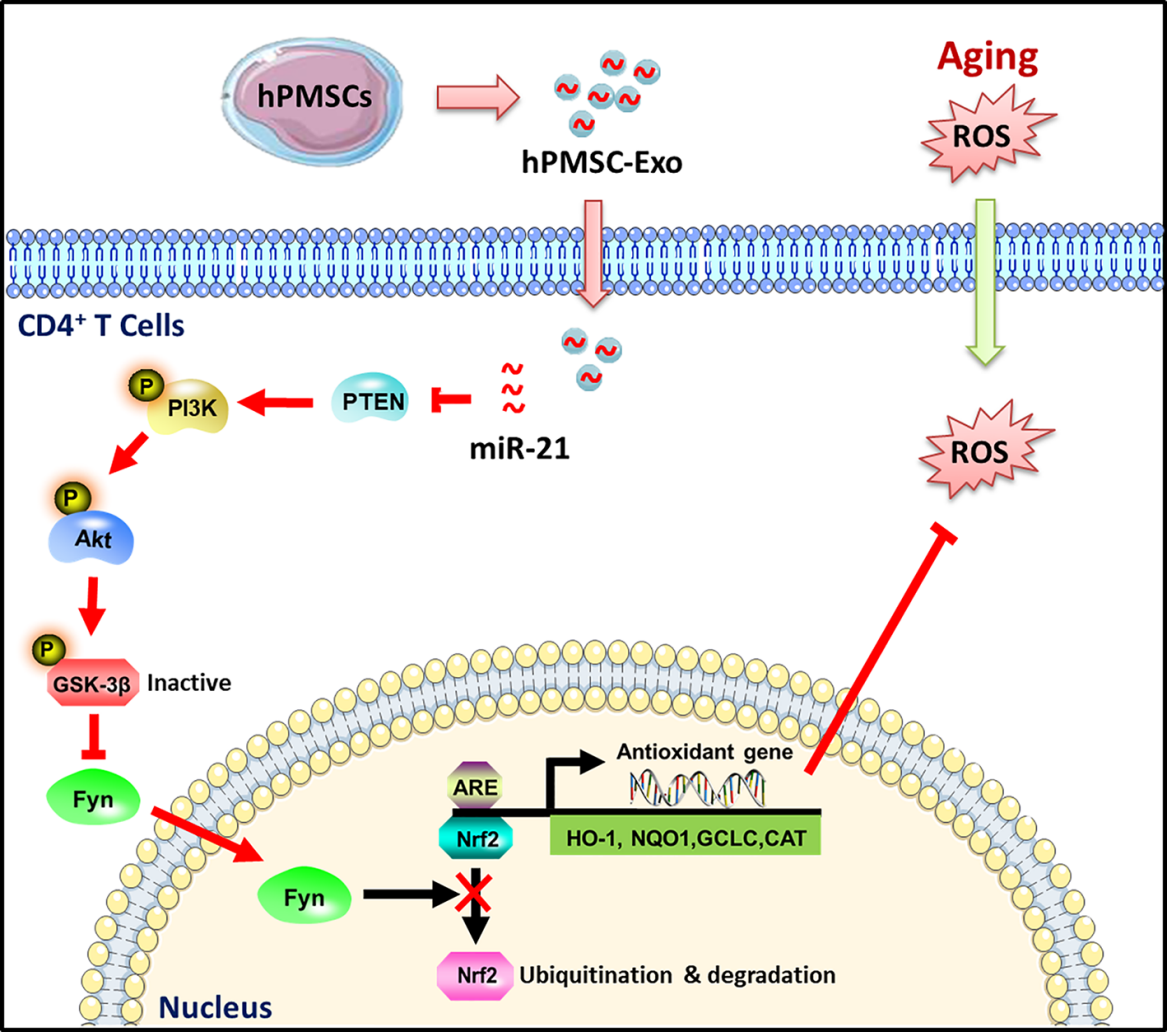
Schematic illustration of the protective effects of hPMSC-Exo on CD4+ T cells senescence. hPMSC-Exo weaken CD4^+^ T cells senescence *via* carrying miRNA-21 and activating PTEN/PI3K-Nrf2 axis mediated exogenous antioxidant defenses.

In conclusion, as illustrated in **Figure 8**, our results indicate a novel role for hPMSC-Exo alleviated D-gal induced CD4^+^ T cells senescence. The attenuation of aging-related oxidative damage was more pronounced with the administration of hPMSC-Exo carrying miRNA-21. The mechanism could be related to the miRNA-21-mediated regulation of the PTEN/PI3K-Nrf2 axis, and the elevation of the antioxidant capacity of senescent CD4^+^ T cells. The findings of the current study provide a novel strategy for the development of a therapeutic method for patients with aging-related immune dysfunction.

## Data Availability Statement

The raw data supporting the conclusions of this article will be made available by the authors, without undue reservation.

## Ethics Statement

The experiments were performed in accordance with protocols approved by the Animal Ethics Committee of Binzhou Medical University.

## Author Contributions

YanliX and XL designed the study. ZY, ZG, LW, YanleX, and YZ collated the data and carried out data analyses. YW, KH, HZ, JZ, and DZ contributed to drafting the manuscript. All authors contributed to the article and approved the submitted version.

## Funding

This investigation was supported by the National Natural Science Foundation of China (No. 32070781); Shandong Provincial Natural Science Foundation (No. ZR2020MH166 and ZR2018QH002); Shandong Province Medical and Health Science and Technology Development Plan Project (No. 2019WS317 and 202003100645); Shandong Province Traditional Chinese Medicine Science and Technology Project (2020Q061).

## Conflict of Interest

The authors declare that the research was conducted in the absence of any commercial or financial relationships that could be construed as a potential conflict of interest.

## Publisher’s Note

All claims expressed in this article are solely those of the authors and do not necessarily represent those of their affiliated organizations, or those of the publisher, the editors and the reviewers. Any product that may be evaluated in this article, or claim that may be made by its manufacturer, is not guaranteed or endorsed by the publisher.
