# Supplementary material for: hPMSCs-Derived Exosomal miRNA-21 Protects Against Aging-Related Oxidative Damage of CD4+ T Cells by Targeting the PTEN/PI3K-Nrf2 Axis
Source: Front Immunol. 2021 Nov 23;12:780897. doi: 10.3389/fimmu.2021.780897 (PMC8649962; doi:10.3389/fimmu.2021.780897)
Supplement: Supplementary file 1 [file Table_1.docx]

| Name | Primer Sequences (Forward/Reverse Primer) |
| --- | --- |
| miR-21 | TAGCTTATCAGACTGATGTTGA  GCCAGCACAGAATTAATACGAC |
| PTEN | AAGACCATAACCCACCACAGC  ACCAGTTCGTCCCTTTCCAG |
| Nrf2 | CAGCATAGAGCAGGACAT  GGAACAGCGGTAGTATCA |
| NQO1 | TTTGAGAGAGTGCTCGTAGC  GGTCTTCTTATTCTGGAAAGG |
| HO-1 | CCCAGTCTATGCCCCACTCT  AGACGCTTTACATAGTGCTG |
| IL-6 | ACATCCTCGACGGCATCTCA  TCACCAGGCAAGTCTCCTCA |
| OPN | TTGCAGCCTTCTCAGCCAA  CAAAAGCAAATCACTGCAATTCTC |
| U6 | CTCGCTTCGGCAGCACA  AACGCTTCACGAATTTGCGT |
| β-actin | CACCACACCTTCTACAATGAG  TACGACCAGAGGCATACAG |

Additional file 1. Sequences used for RT-qPCR
